# Veterinary Medicine in the United States

**Published:** 1884-04

**Authors:** 


### EDITORIAL DEPARTMENT.

### VETERINARY MEDICINE IN THE UNITED STATES.

IN our last issue we endeavored to show why true science does not flourish in connection with the medical institutions of our country, and concluded our remarks with what we considered to be the chief work of a Veterinary School.

Our readers will bear in mind that it is absolutely true that the work of the best type of Veterinary Schools is not by any means of an educational character, but of a scientific nature, that is, an institution for research. In so far as this element of research is wanting in a medical school, in so much is it a failure; hence, from this standpoint, every Veterinary School at present in operation in this country is a failure, and the majority of the medical schools; in fact, none of them are what we have a right to demand.

A very mistaken idea is often urged against this argument, i. e., that *this is a young country*, and hence we cannot rightly expect that we should attain the same degree of perfection in these things that has been obtained in European countries. As said above, this is an error. Such questions are not, or should not, be judged from this standpoint, but from the ability, that is the wealth of a country, to support scientific research, and in this regard we are second to no nation in the world. Another reason, which we stated in our last, is that it is also dependent upon the intelligence of the masses, or more particularly the ruling class in a country, and though it is true, this is a disgraceful and should be a mortifying reflection upon the American people.

We cannot refrain from asserting that we think, in this country, that *inheritance* has much to do with it. The arguments that we are still a "young country," and that inheritance has much to do with our sad condition, find proof in the conditions in Britain, a country as old as most of the Continental nations, and as wealthy, yet, as we have shown, science has no home there. We make bold to assert that were the ruling influence here of a German, instead of an English tendency, that we should see science flourishing under the protection of the spangled banner of the United States.

It is not with this side of the question that we have to do at present, however. The points which we are about to place before our readers are especially for the consideration of American Veterinarians.

We have said that *no American Veterinary School is what it should be, or what is necessary to fulfill the demands that the people have a right to make upon it.* We might go further and say *that no contemplated school will answer this purpose; we can truly say it will not as at present endowed or conducted.*

This is no reflection upon the graduates of our schools. If the opportunity is not given them to obtain a scientific education in this country they cannot be expected to have it, and the man is a fool who, at the present time, would go to Europe and spend his time and money to obtain it. To be sure there is a certain personal gratification and amusement in being able to gratify such tastes even from the literary point of view, but if a man has them, he will find that he is like a starving man on a barren island, with another bountifully supplied with food in view, but which he cannot reach; he sees all around him material and opportunity to work, but, for want of means, both mind and hands are tied; and he also finds his scientific tendencies instead of helping him, harm in practice, and that he is no better appreciated by the public than men of far inferior abilities, for it is only exceptionally that cases occur in practice demanding all one's scientific acumen and practical ability. Human surgery has the advantage of us in this way. The title Veterinary Surgeon is a misnomer in our opinion.

With every respect for our colleagues in practice, we can but feel that every Veterinary School in this country is at present more or less injurious to the nation. They are private business speculations, without a strictly moral, professional, or strong financial basis. We do not think there is at present the need of the number of practitioners that many claim. There is no work of them, but, still more, we fail in receiving that stimulation by which alone Veterinary medicine can be advanced. We allude to the inducements offered to men of superior attainments where a well organized Veterinary Police system exists in a country.

The evil results of the present system of incorporating private schools must be manifest to every reflecting member of our profession.

The manner in which the schools are conducted makes this all too evident.

First they advertise for students, offering every inducement possible to obtain them, in the same way that business houses advertise their goods. One school says "*it has more graduates in successful practice than any other on the Continent,*" but not a word do we know about the terms of graduation, or how easy it is to slip through the session of this school and obtain the desired diploma.

Another claims to be the only school in this country authorized to "confer a degree in Veterinary Science (?)" as may be seen from the following :

"ANNOUNCEMENT OF THE SESSION OF 1883–1884.

The College, by an act of the legislature, passed in 1857, is empowered to confer the degree of Veterinary Surgeon, and is the only chartered institution specially devoted to veterinary education in the United States.

"Veterinary Departments have been added to some of our Universities, namely : Cornell, Pennsylvania and Harvard, but no other institutions in the United States have the power, to our knowledge, to confer a degree in Veterinary Science."

The injurious influence of such an announcement is seen in the fact that this school is noticed in a late number of the *Veterinary Journal* of Vienna *as the only legally qualified school in the United States.*

Such an "announcement" infers that the holders of the diplomas of other American schools are illegally qualified as Veterinarians. It matters not to us how they are incorporated; they are, in some way; the diploma of one is just as good as that of another, unless really fraudulent affairs, and dealers in such parchments, and the holders of them are as legally entitled by American laws to public appreciation as those who hold the certificates of a Continental School or that of the Royal College of Veterinary Surgeons.

But the result is much worse than the action of these schools at first appears.

The holders of the parchments each claim to have some educational superiority over others, which leads to professional jealousy and prevents any unity of purpose in the profession.

We are a "house divided against itself." We see the West opposing the East, and no union among the graduates in any single State. Professional enthusiasm is as dead as a cadaver upon the dissection table. A comic monthly devoted to what (?) is started in the West as a "National Veterinary Journal;" then follows the incorporation of a veterinary school, and then State organizations with said monthly *as their organ*, and finally "a National Veterinary Association," which is so only in name, and is a distinct Western organization; in opposition to which we have the older American Veterinary Association, an Eastern organization, which has, through unfortunate and undesired circumstances, become, in reality, almost entirely an association composed of the graduates of the American Veterinary College. The last has done no work that has advanced our profession, and we may be pretty sure our Western sister will only bring forth still-born young, if she conceives at all.

The "announcement" previously alluded to speaks of the universities of Pennsylvania, Cornell and Harvard as having veterinary departments, and infers that they have no authority to grant veterinary diplomas.

As to Harvard we think this is true, for we know of no legislative act of incorporation or authority given Harvard to grant diplomas, except it be an assumed right based upon its general charter. No one will, however, question the value of its diploma, especially in New England.

But to veterinarians we have a word to say with reference to Harvard, and that is that every one of us is professionally bound to do our utmost to prevent students from going there. The adoption of the "subscription plan," by which the Harvard Hospital is conducted, and which results in a disgraceful cutting of prices for services rendered, should be known to every veterinarian. We have no words to express our personal indignation at the conduct of the advisers of Harvard in this matter, and it is a disgrace to our profession that any members of it would accept a position in connection with such a school. The History of American Veterinary Medicine is as yet in its infancy, but when it comes to be written we know what the verdict pronounced upon the action of Harvard and its veterinary advisers will be. It is recorded already for its prototype in London, and we feel like bowing our head with shame that such an abortion should be planted on American soil. It is not the action of Harvard as such, could it be confined to that institution, that we have so much to fear, but the iufluence of this college is great over the land; it has its graduates in the legislative halls of every State in this Union, and when other States or institutions come to incorporate veterinary schools we have to dread that this example will be followed, and this, worse than the Rinderpest, become domesticated on American soil, and, like witch-grass, send its demoralizing roots over the land.

Every Veterinarian in this country has, however, much occasion for rejoicing, for we have been assured that the Veterinary Department of the University of Pennyslvania, which was at first inaugurated under this subscription plan, will not open with it, but will be conducted on the most stringent principles of medical ethics. Dr. Huidekoper, the Dean of this Department, writes: "*I, like you, am absolutely against any 'subscription plan,' and expect to run a large hospital and clinic without it. The entire Veterinary profession here is interested in our undertaking; graduates and reputable empirics, the latter have already enrolled their sons or brothers as students.*" We commend the above to the consideration of Harvard. There is not a Veterinarian in Massachusetts, except the two at this school, but indignantly condemns its action; not a medical doctor that is not hide and brain bound that is not opposed to the course adopted by Harvard in conducting its school.

It is said that we are sacrilegiously attacking an old, revered, and time-honored institution, to which we answer, *that nothing is sacred but the truth, nothing worthy of reverence but the right, which will stand criticism till the everlasting hills sink out of sight.*

With such conditions as these it is no wonder that professional unity is wanting among Veterinarians. Yet to progress and improve we must have it. Is the profession dead to its own duty? or is it only slumbering from indolence? The questions we are discussing are vital ones. The columns of this Journal are open to every man that has something to say worth reading. Must it all be done editorially? We know that many of our colleagues are with us. They should remember that *the pen is the sword of to-day ;* that intellect wins ; that it is said we have few men that are able writers and thinkers in our profession. We have more than the world knows ; laziness is their chief fault, and professional indifference the second.

The necessity of a national Veterinary Police system is becoming more prominent every day. Without its stimulating effect our profession has never advanced in any country. The Veterinary practitioner, per se, *has never advanced our profession an iota through success in practice.* The Veterinary Police official, as the executor of the teachings of the school scientist, has.

Without a proper recognition by the Government of its duties to the people this end cannot be attained.

Thus our work is indicated. In every way, by advocacy in public and private, every true professional must endeavor to bring about this end.

We have been unfortunate in the men selected by the Government to do its work. They have failed utterly in professional responsibility. They have been the tools of their political employers, not their advisers as they should have been. At present unless a man can be made available, or is willing to be disgracefully subservient to politicians, he has no chance at Washington. United millions have been lost from contagious and infectious animal diseases ; thousands of dollars flittered away by politicians and their employees, and what has been the result? Nothing !

The agriculturalists and stock-raisers, great and small, must be either wofully ignorant or terribly blind to their own interests if they allow this condition to exist much longer with the remedy in their own hands.

What we want at Washington is a technical deputation or Commission, as a permanent post of the government, to control the subject of animal diseases and animal hygiene, composed of the best men that can be got, that are not politicians, from among our agriculturists—an eminent lawyer and a veterinarian that loves his country and profession better than himself.

These remarks have been called forth by the perusal of the last Report of the Department of Agriculture on the "Contagious Diseases of Domestic Animals," a book of 251 pages.

We cordially recommend it to every veterinarian in the country. The part contributed by Dr. Salmon is worthy of diligent study and profound reflection. It shows that as far as an investigator goes we have the right man in the right place, a credit to our profession and himself; and the government should not fail in supplying the laboratory and means to continue the work he seems so ably fitted for.

The work is largely devoted to one disease, *the Texas or Southern Cattle Disease.* When we ask what have been the results of all this study and investigation, aside from the money spent, we discover that but one fact has been established, viz.: *that this disease* is gradually extended northward, and that Southern animals have the faculty of impregnating lands upon which they have grazed until, after long continuance of such a procedure, these lands become centres of infection to Northern cattle that are brought upon them.

The disease has not yet been produced by inoculation, though this result may be hoped for by continued experimentation. Dr. Salmon has found in the spleen a peculiar germ of the dumb-bell variety, diplococcus, which he could not find either in the blood or other organs. This germ is capable of cultivation, and we share the hopes of the Doctor that it may be *the cause*, though we are not as enthusiastic as he is upon the future results of diluted inficientia as a means of prevention for all contagio-infectious diseases. Dr. Delmer was sent to Texas and vicinity to study the disease there, but has added nothing to our knowledge. Our space limits us in further consideration of this contribution to veterinary literature.

We can but think that much more could and ought to have been done. This is, in reality, *not a report upon the* "*Contagious Diseases of Domesticated Animals,*" but almost entirely upon one disease. A true and accurate report will never be issued until the Government learns to make use of the whole Veterinary profession, and organizes a Veterinary Police System. B.

